# Association of N-Linked Glycoprotein Acetyls and Colorectal Cancer Incidence and Mortality

**DOI:** 10.1371/journal.pone.0165615

**Published:** 2016-11-30

**Authors:** Paulette D. Chandler, Akintunde O. Akinkuolie, Deirdre K. Tobias, Patrick R. Lawler, Chungying Li, M. Vinayaga Moorthy, Lu Wang, Daniel A. Duprez, David R. Jacobs, Robert J. Glynn, James Otvos, Margery A. Connelly, Wendy S. Post, Paul M. Ridker, JoAnn E. Manson, Julie E. Buring, I-Min Lee, Samia Mora

**Affiliations:** 1 Division of Preventive Medicine, Brigham and Women’s Hospital, Harvard Medical School, Boston, Massachusetts, United States of America; 2 Division of Cardiovascular Medicine, Brigham and Women’s Hospital, Harvard Medical School, Boston, Massachusetts, United States of America; 3 Department of Epidemiology, Harvard T.H. Chan School of Public Health, Boston, Massachusetts, United States of America; 4 Department of Medicine, University of Minnesota, Minneapolis, Minnesota, United States of America; 5 Division of Epidemiology and Community Health, School of Public Health, University of Minnesota, Minneapolis, Minnesota, United States of America; 6 LabCorp, Raleigh, North Carolina, United States of America; 7 Division of Cardiology, Department of Medicine, Johns Hopkins University School of Medicine, Baltimore, Maryland, United States of America; 8 Department of Epidemiology, Bloomberg School of Public Health, Johns Hopkins University, Baltimore, Maryland, United States of America; West China Second Hospital, Sichuan University, CHINA

## Abstract

**Background:**

Acute phase proteins highlight the dynamic interaction between inflammation and oncogenesis. GlycA, a novel nuclear magnetic resonance (NMR) inflammatory marker that identifies primarily circulating N-acetyl glycan groups attached to acute phase proteins, may be a future CRC risk biomarker.

**Methods:**

We examined the association between GlycA and incident CRC and mortality in two prospective cohorts (N = 34,320); Discovery cohort: 27,495 participants from Women's Health Study (WHS); Replication cohort: 6,784 participants from Multi-Ethnic Study of Atherosclerosis (MESA). Multivariable Cox models were adjusted for clinical risk factors and compared GlycA to acute phase proteins (high-sensitivity C-reactive protein [hsCRP], fibrinogen, and soluble intercellular adhesion molecule-1 [sICAM-1]).

**Results:**

In WHS (median follow-up 19 years, 337 cases, 103 deaths), adjusted HRs (95% CIs) per SD increment of GlycA for CRC incidence and mortality were 1.19 (1.06–1.35; p = 0.004) and 1.24 (1.00–1.55; p = 0.05), respectively. We replicated findings in MESA (median follow-up 11 years, 70 cases, 23 deaths); HRs (95% CIs) per SD of GlycA for CRC incidence and mortality were 1.32 (1.06–1.65; p = 0.01) and 1.54 (1.06–2.23; p = 0.02), respectively, adjusting for age, sex, and race. Pooled analysis, adjusted HR (95% CI) per SD of GlycA for CRC incidence and mortality was 1.26 (1.15–1.39; p = 1 x 10^−6^). Other acute phase proteins (hsCRP, fibrinogen, and sICAM-1) had weaker or no association with CRC incidence, while only fibrinogen and GlycA were associated with CRC mortality.

**Conclusions:**

The clinical utility of GlycA to personalize CRC therapies or prevention warrants further study.

**Trial Registration:**

ClinicalTrials.gov: WHS NCT00000479, MESA NCT00005487

## Introduction

The emerging field of acute phase proteins as cancer biomarkers[1] highlights the dynamic interaction between inflammation and tumor cells.[2] Acute phase proteins play a key role in chronic inflammation, and regulate complex changes in the tumor microenvironment such as angiogenesis[3] and proliferation.[4] GlycA, a novel marker of inflammation measured by targeted metabolomics using nuclear magnetic resonance (NMR) spectroscopy, identifies N-acetyl glycan groups (Fig A in S1 File) mostly attached to acute phase glycoproteins (predominantly α1-acid glycoprotein [orosomucoid], haptoglobin, α1-antitrypsin, α1-antichymotrypsin, and transferrin).[5] C-reactive protein (CRP), an acute phase protein that does not contribute to the GlycA signal, as well as the acute phase proteins that do contribute (α1-acid glycoprotein, haptoglobin, α1-antitrypsin, α1-antichymotrypsin, and transferrin), have differential glycosylation patterns that have been linked to distinct cancer types including CRC and stages of malignancy.[6–8] These glycosylation signatures may be useful biochemical tumor markers for initial diagnosis, staging and monitoring of colorectal cancer.[9]

To date, no established inflammatory biomarker has been consistently associated with incident colorectal cancer.[10, 11] Prospective studies have evaluated pre-diagnostic circulating CRP levels and CRC risk, but with inconsistent results.[12] Currently, carcinoembryonic antigen (CEA), also a glycoprotein, is the crucial biomarker for monitoring CRC recurrence and prognosis.[13] [14] The combination of CEA and the glycosylated acute phase proteins (haptoglobin, α1-antitrypsin, and α1-acid glycoprotein) was more strongly associated with CRC progression than CEA alone in CRC patients receiving chemotherapy.[15] CEA has low specificity for CRC, thereby limiting its usefulness for identifying incident CRC.[16] With the limited clinical applicability of CEA, additional candidates are needed as CRC risk markers. Quantifying and defining the human glycome in CRC has received interest as a novel tool to identify markers of CRC and potential mechanistic mediators of oncogenesis. [17] [18, 19] Hence, we hypothesized that GlycA, a novel systemic inflammatory biomarker of protein glycan N-acetyl groups, is related to incident colorectal cancer and mortality. Further, we compared the CRC cancer and mortality risk associated with GlycA with other circulating acute phase proteins, high-sensitivity C-reactive protein (hsCRP), fibrinogen, and soluble intracellular adhesion molecule 1 (sICAM-1).

## Materials and Methods

### Discovery Study Population

The discovery study population was derived from the Women’s Health Study (WHS, n = 39,876), a completed randomized controlled 2x2x2 factorial trial of aspirin, β-carotene, or vitamin E versus placebo in the primary prevention of cancer and cardiovascular disease.[20, 21] Women were healthcare professionals, ≥45 years old, and free of cancer and cardiovascular disease at study entry (1992–1996). After trial completion, extended post-trial follow-up of participants remained on-going with follow-up reported herein through 2013. Of the 39,876 randomized women in the trial, 28,345 (71%) provided a baseline blood sample. The study was approved by the Human Subjects Committee at the Brigham and Women's Hospital, Boston, MA. Additional information about the study population is provided in the S1 File.

### Replication Cohort

We evaluated the associations found in WHS in an independent multiethnic cohort of men and women from the Multi-Ethnic Study of Atherosclerosis (MESA).[22] MESA was chosen because GlycA levels were already measured in MESA to evaluate the association between GlycA and cardiovascular disease. Briefly, this community-based study enrolled 6,814 men and women, ages 45–84 years, of African-American (28%), Hispanic (22%), White (38%), and Chinese-American (12%) ethnicity, free of self-reported active treatment of cancer and cardiovascular disease at baseline entry (2000–2002). The study was approved by the institutional review boards of the participating institutions, and subjects gave written informed consent.[22] Standardized questionnaires and procedures were used to determine age, sex, ethnicity, and clinical features.[22] GlycA was measured at baseline among 6,784 of the 6,814 participants.

### Statistical analyses

Baseline characteristics of participants across quartiles of GlycA were summarized as means (standard deviation [SD]), or medians (25^th^ to 75^th^ percentiles) for quantitative variables, and as percentages for qualitative variables. GlycA has a normal distribution from the spectral deconvolution algorithm used to quantify GlycA signal. Comparisons were statistically assessed with the Wilcoxon rank sum and χ^2^ tests. Spearman coefficients were used to correlate GlycA with risk factors and inflammatory biomarkers. Person-years of follow-up and rates were calculated, and cumulative incidence was obtained according to quartiles of GlycA and log-rank test was used to compare curves. Hazard ratios (HRs) and 95% confidence intervals (CIs) of incident CRC events and mortality were calculated from Cox-proportional hazard regression for mid-quartile scores and per SD increment. Exposure time was calculated as the time from enrollment to incidence/death or censoring. In the initial WHS analysis, incident CRC cases only include nonfatal CRC to be consistent with prior WHS analyses. However, for the pooled analysis of WHS and MESA, WHS CRC cases included fatal CRC cases. As there was no significant interaction between CRC, GlycA, and randomization arms (including aspirin), the groups were pooled and indicators of the randomized treatments were included as covariables. SAS version 9.4 (SAS Institute, Cary, NC, USA) was used for all analyses except the pooled analysis (STATA version 14, College Station, TX).

Adjustment for potential confounders or mediators was completed with sequential models. Additional information about the models is provided in the S1 File. P for trend was calculated across quartiles for WHS and tertiles for MESA (given smaller number of cases, n = 70 cases). The longitudinal CRC incidence associated with GlycA in WHS did not violate the proportional hazards assumption (p value = 0.62 for test of proportional hazard assumption in a model assuming linearity of GlycA). To examine the possibility of reverse causation, we performed sensitivity analysis excluding CRC cases occurring during the first 2 years and repeated this after excluding CRC cases occurring within the first 5 years. We compared the CRC cancer incidence and mortality risks associated with GlycA to that of other established systemic inflammatory biomarkers (ln hsCRP, fibrinogen, and sICAM-1).

Following replication in MESA, the study specific estimates were combined in a pooled analysis and pooled into Forest plots using random effects models to account for inter-study heterogeneity. For the CRC pooled analysis, WHS incidence cases included fatal and nonfatal cases and MESA incidence cases included fatal and nonfatal cases. Additional information about the analysis plan is provided in the S1 File. All analyses were specified *a priori* by the academic investigators except where explicitly indicated. WHS and MESA are registered at ClinicalTrials.gov: WHS NCT00000479 and MESA NCT00005487, respectively.

## Results and Discussion

### WHS

The mean age (SD) of the WHS cohort at baseline was 54.7 (7.1) years. Stratification by GlycA quartiles identified a higher prevalence of CRC risk factors (e.g. BMI) among those with higher levels of GlycA (Table 1).

**Table 1:** Baseline characteristics of WHS participants by quartiles of GlycA ^a^.

	Quartile 1	Quartile 2	Quartile 3	Quartile 4
	≤326 μmol/L	327–369 μmol/L	370–416 μmol/L	>416 μmol/L
N	6992	6791	6865	6847
Age, years	51(48, 57)	53 (49, 59)	54 (50, 60)	54 (50, 60)
Race/ethnicity, %				
Caucasian	93.7	94.8	95.1	94.5
Hispanic	1.0	1.2	1.1	1.0
Black	1.7	1.7	1.6	2.4
Asian/Pacific Islander	2.5	1.3	1.1	0.6
American Indian/Alaskan Native	0.3	0.3	0.5	0.5
Postmenopausal, %	45.9	53.8	57.5	60.5
Randomized aspirin, %^b^	50.3	48.3	50.7	50.7
Randomized vitamin E, %^c^	49.9	51.1	49.3	49.9
Randomized beta carotene, %^c^	49.6	50.0	49.3	50.3
Body mass index, kg/m^2^ mean (SD)	23.6 (3.5)	25.1 (4.2)	26.5 (4.7)	28.7 (5.7)
Physical activity, MET-hrs/week, mean (SD)	17.8 (20.8)	15.9 (19.5)	13.5 (16.7)	11.7 (15.5)
Smoking, %				
Current	7.7	10.6	12.6	15.8
Past	36.7	37.6	36.9	35.4
Never	55.6	51.8	50.3	48.7
Family history of CRC, %^c^	10.0	10.7	10.6	10.3
History of polyps, %^d^	2.1	2.7	2.7	2.7
Fruit and vegetable intake, servings/d, mean (SD)^d^	6.2 (3.9)	6.2 (3.5)	6.1 (3.4)	6.0 (3.6)
Fiber intake, g/d, mean (SD)	19.4 (8.4)	19.1 (8.0)	19.1 (8.3)	18.7 (8.0)
Red meat, servings/d, mean (SD)	0.7 (0.5)	0.7 (0.6)	0.7 (0.6)	0.8 (0.6)
Dietary calcium, mg/d, mean (SD)^b^	794.1(357.0)	789.7 (357.7)	792.6 (359.8)	775.9 (355.2)
Alcohol, g/d, mean (SD)	4.7 (8.1)	4.5 (8.6)	4.0 (8.2)	3.3 (8.0)
Total calories, kcal/d, mean (SD)^c^	1718 (515.5)	1726 (524.0)	1737 (531)	1738 (545)
Multivitamin use, %^c^	86.2	85.4	86.3	85.2
Lipid lowering medications, %	1.2	2.3	3.2	6.0
Postmenopausal hormone use- current, %	35.5	40.8	45.3	49.0
Diabetes, %	1.0	1.5	2.1	4.9
Hemoglobin A1c, %	4.9 (4.8, 5.1)	5.0 (4.8, 5.2)	5.0 (4.9, 5.2)	5.1 (4.9, 5.3)
hsCRP, mg/L	0.7 (0.4, 1.5)	1.5 (0.7, 2.9)	2.6 (1.4,4.6)	5.1 (2.8, 8.3)
sICAM, ng/mL	317 (281, 360)	337 (297,383)	349 (308, 400)	373 (328, 430)
Fibrinogen, mg/dL	314 (279, 350)	341 (304, 384)	363 (321, 409)	402 (353, 457)

Data presented as median (25^th^ quartile, 75^th^ quartile) unless otherwise indicated.

Abbreviations: BMI = body mass index, MET-hrs/wk = metabolic equivalent hours per week, hsCRP = high sensitivity C-reactive protein, sICAM-1 = soluble intracellular adhesion molecule 1.

^a^ P value <0.0001 unless otherwise indicated.

^b^ P value = 0.01

^c^ P value >0.05

^d^ 0.01<P value <0.05

Likewise, levels of hsCRP, sICAM-1, and fibrinogen were higher by increasing quartiles of GlycA, and correlated moderately with GlycA with the strongest correlation for hsCRP (Spearman correlation coefficients 0.30 to 0.61; Table A in S1 File).

### Incident CRC and Mortality

**Fig 1:**
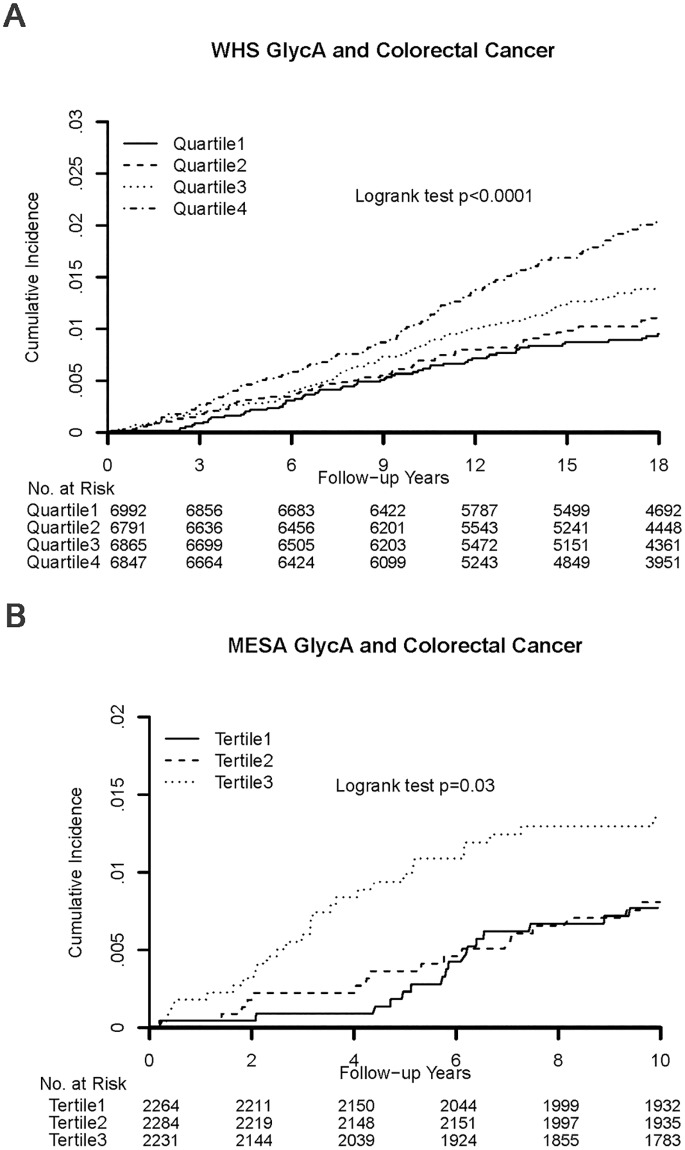
Colorectal Cancer Cumulative Incidence According to GlycA Quartile in WHS (a); Colorectal Cancer Cumulative Incidence According to MESA GlycA Tertiles (b).

Over a median follow-up of 19 years among the 27,495 WHS participants (Fig B in S1 File), 337 incident CRC cases and 103 CRC deaths occurred. For WHS, incident CRC cases only include nonfatal CRC. Cumulative incidence curves for CRC events (adjusted for age) diverged according to quartiles of GlycA (Fig 1, p for log-rank<0.0001; Table 2).

**Table 2:** 

GlycA	Quartile 1	Quartile 2	Quartile 3	Quartile 4	P for trend	Per SD*	P Value
**Range, μmol/L**	≤326	327–369	370–416	>416			
**Incident CRC, N cases/total**	61/6992	70/6791	86/6865	120/6847			
**Incident Rate per 1,000 person-years**	0.52	0.62	0.76	1.09			
**CRC deaths, N cases/total**	19/6992	21/6791	26/6865	37/6847			
**Death Rate per 1,000 person-years**	0.15	0.17	0.21	0.31			
**Incident CRC, model 1**	1.0 (ref)	1.08 (0.77–1.53)	1.29 (0.93–1.79)	1.83 (1.35–2.50)	<0.0001	1.26 (1.13–1.39)	<0.0001
**Incident CRC, model 2**	1.0 (ref)	1.05 (0.73–1.51)	1.23 (0.87–1.75)	1.55 (1.09–2.20)	0.006	1.19 (1.06–1.35)	0.004
**CRC death, model 1**	1.0 (ref)	1.01 (0.54–1.88)	1.21 (0.67–2.19)	1.74 (1.00–3.03)	0.02	1.29 (1.07–1.55)	0.008
**CRC death, model 2**	1.0 (ref)	0.92 (0.47–1.80)	1.26 (0.67–2.36)	1.46 (0.77–2.76)	0.15	1.24 (1.00–1.55)	0.05

* Model 1 Hazard ratio from Cox regression models adjusted for age and trial treatment assignment

Model 2 Hazard ratio from Cox regression models adjusted for age, trial treatment assignment, race, family history of colorectal cancer, alcohol, exercise, smoking, menopausal status, postmenopausal hormone use; alternative healthy eating index, multivitamin use; intake of red meat, vegetables and fruits, supplemental and dietary calcium, fiber, total calories, history of polyps, body mass index, and hemoglobin A1c.

The risk factor-adjusted model 2 HR (95% CI) per SD (68.4 μmol/L) higher GlycA for CRC incidence was 1.19 (1.06–1.35; p = 0.004) and CRC mortality 1.24 (1.00–1.55; p = 0.05) (Table 2). CRC incidence and mortality increased by quartiles of GlycA with minimal attenuation after adjustment for clinical variables.

### MESA Replication

Among 6,784 MESA participants (median follow-up: 11 years) 70 incident CRC cases and 23 CRC deaths occurred (Fig 1).

Compared with WHS, MESA participants were older (mean [SD] age of 62.2 (10.2)) and were 47.2% male (Table B in S1 File). CRC incidence and mortality were increased per SD (62 μmol/L) of GlycA with similar magnitudes of association as in the WHS although these associations were no longer significant in Model 2 after accounting for clinical risk factors, HR (95%CI) were 1.21 (0.95–1.55; p = 0.12) and 1.34 (0.88–2.03; p = 0.17) respectively. (Table C in S1 File)

### Evidence of GlycA-CRC Risk beyond the Other Acute Phase Proteins

Examination of hsCRP, sICAM-1, and fibrinogen with CRC risk factor-adjusted model 2 per SD yielded no significant associations with CRC incidence or mortality with any of the systemic inflammatory biomarkers with the exception of fibrinogen (Table D in S1 File). Fibrinogen was significantly associated with increased risk of CRC death in quartile analysis Q1to Q4, 1.0 (ref), 0.97 (0.45–2.11), 1.79 (0.90–3.57), 1.81 (0.90–3.66), p for trend = 0.04 but not for per 1 SD.

Then we performed sensitivity analyses to investigate the potential risk associated with GlycA beyond the known markers of systemic inflammation (Table E in S1 File). Incident CRC risk associated with GlycA remained significant after additionally adjusting for any of the three systemic inflammatory biomarkers (hsCRP, fibrinogen, or sICAM-1).) with slightly increased magnitudes of association: HR (95%CI) of incident CRC per SD of GlycA after adjusting model 2 for either fibrinogen, hsCRP, or sICAM were: 1.22 (1.07–1.39; p = 0.003); 1.27 (1.10–1.45; p = 0.0009); and 1.20 (1.06–1.36; p = 0.004) respectively. Magnitudes of association also remained robust in quartile analysis. (Table E in S1 File) Results were similar when CRC death was examined.

### Stratification by CRC risk factors

**Fig 2:**
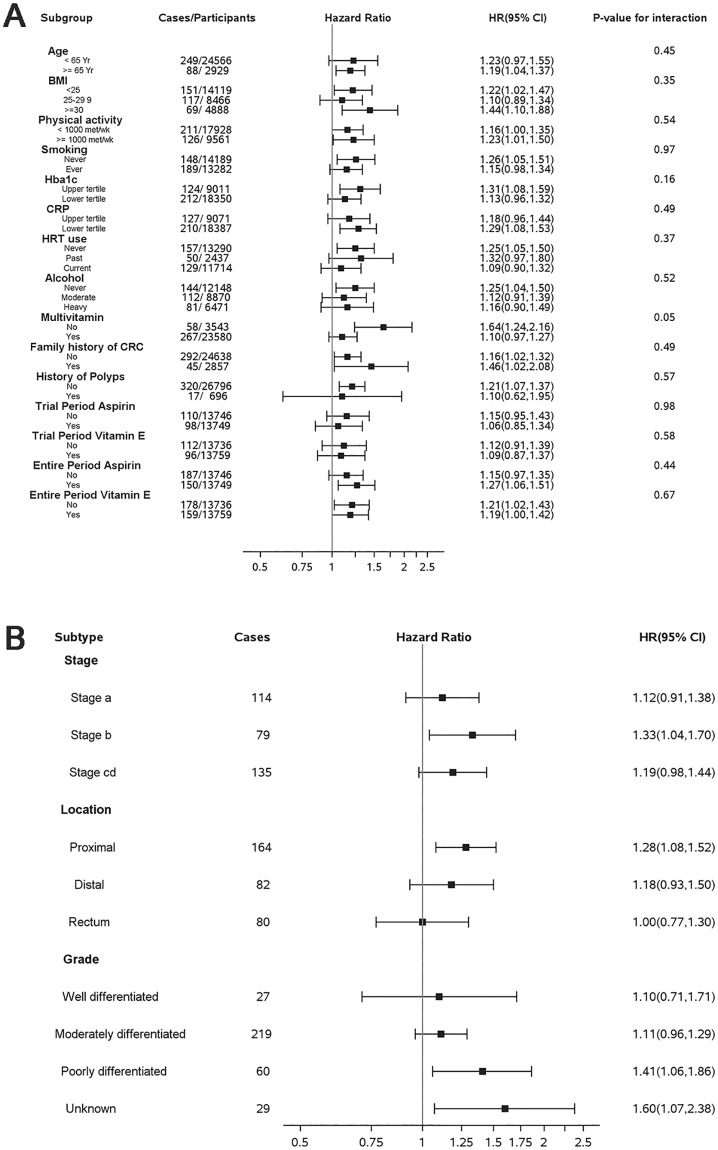
Hazard Ratios and 95% confidence intervals per GlycA SD for Incident Colorectcal Cancer in Subgroups (a); Hazard Ratios and 95% confidence intervals per GlycA SD for Incident Colorectal Cancer Tumor Characteristics (Stage, Location, Grade) (b).

In WHS analyses stratified by CRC risk factors such as age, BMI, increasing GlycA remained associated with increased risk of incident CRC with no evidence of effect modification by the established CRC risk factors (p for interaction ≥ 0.06 for all subgroups except multivitamin subgroup p for interaction = 0.05) (Fig 2).

### Sensitivity Analysis by Follow-Up Time

WHS sensitivity analyses were done to examine potential reverse causation between GlycA and CRC. With exclusion of the first 2 years of follow-up, model 2 HR (95% CI) per SD higher GlycA was 1.18 (1.04–1.34; p = 0.009). Excluding the first 5 years of follow-up, HR and 95% CI per SD was 1.20 (1.05–1.39; p = 0.01). The observed point-estimates were also similar for the association between GlycA and incident CRC and mortality during the aspirin and vitamin E trial 10-year treatment period (208 CRC cases and 51 deaths, data not shown).

### Associations with tumor characteristics

In WHS exploratory analyses, some CRC tumor characteristics were significantly associated with GlycA (Fig 2), including higher Duke stage, proximal tumor location, and less differentiated tumors.

### Incident Colorectal Cancer in Subgroups

Subgroup multivariate hazard ratios (HRs) for incident colorectal cancer were adjusted for trial treatment assignment, age, race, family history of colorectal cancer, alcohol, exercise, smoking, post menopausal versus premenopausal, postmenopausal hormone use-never, past, current; alternative healthy eating index continuous, multivitamin yes/no current use; red meat intake servings/d, total vegetable and fruits intake servings/day, supplemental calcium, dietary calcium, fiber grams/day, total calories, history of polyps, randomized aspirin use during trial period, randomized vitamin E use during trial period, entire follow-up period for aspirin arm, entire follow-up period for vitamin E arm.

### Pooled Analysis

**Fig 3:**
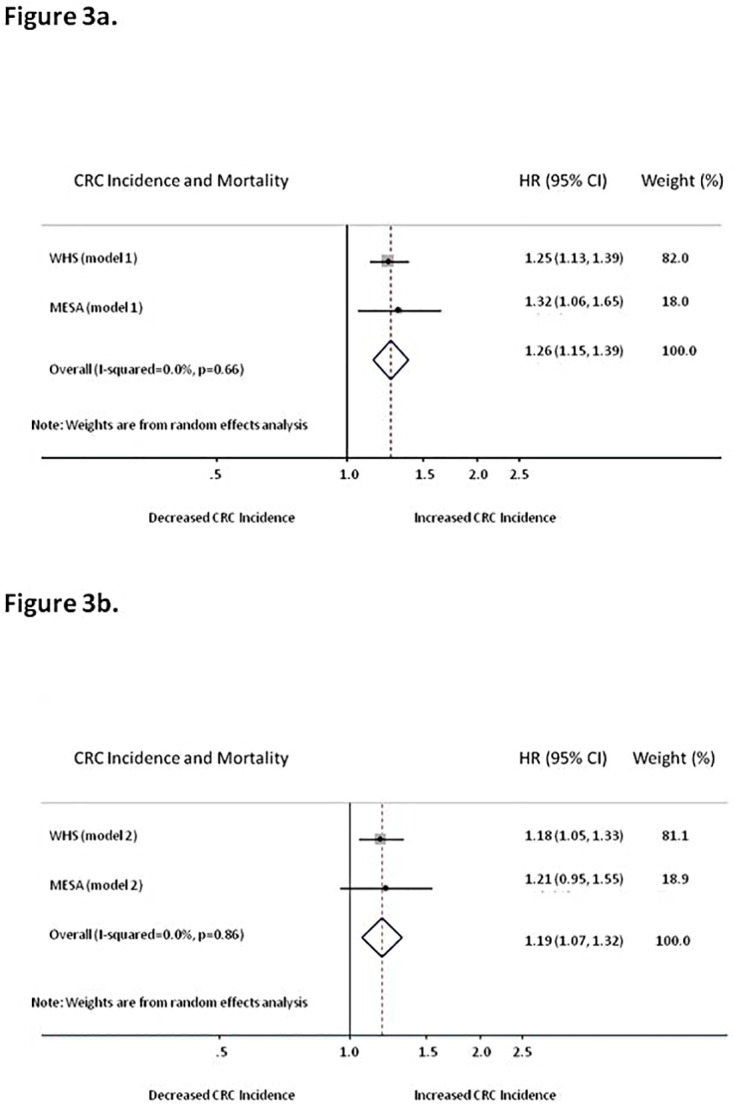
Pooled Associations between GlycA and Colorectal Cancer Incidence and Mortality.

From random effects pooled analysis of the CRC incidence and mortality in the WHS and MESA cohorts per SD increment in GlycA, the pooled model 1 HR (95% CI) per SD for CRC incidence and mortality was 1.26 (1.15–1.39; p = 1 x 10^−6^) with no significant heterogeneity (I-squared = 0% p = 0.66), with similar results for the more fully adjusted model 2 (Fig 3).

Glycosylation of acute phase proteins in cancer yields specific glycoforms of specific glycoproteins and may provide useful tumor markers.[23] This study examined the longitudinal association between an NMR-measured plasma summary biomarker of circulating N-acetyl glycans on acute phase proteins and CRC incidence and mortality among initially healthy individuals. Using data on 27,495 initially healthy women with over 19 years of follow-up in the WHS, GlycA, an aggregate of circulating N-acetyl groups of N-acetylglucosamine and N-acetylgalactosamine glycan moieties, was significantly associated with CRC incidence and mortality. The CRC incidence and mortality findings were replicated in an independent multi-ethnic cohort of 6,784 men and women from the MESA study. To our knowledge, no other study has longitudinally evaluated the association of a glycan based inflammatory biomarker and incident CRC in individuals free of cancer at baseline. WHS sensitivity analyses excluding the first 2 or 5 years emphasize the absence of reverse causation. The robust association between GlycA and CRC incidence and mortality suggests that glycosylation changes may contribute the role of inflammation on CRC carcinogenesis.

A prior study demonstrated that GlycA levels are chronically elevated for over a decade and associated with a myriad of inflammatory cytokines.[24] Although individuals with elevated GlycA were more likely to have risk factors associated with CRC, CRC incidence remained significantly associated with increased GlycA after adjusting for clinical risk factors. GlycA correlated with established acute phase proteins, but was an important predictor of CRC incidence and mortality after adjustment for these biomarkers. Overall, these results suggest a robust association with a measure of circulating N-linked glycoprotein acetyls (predominantly on acute phase proteins) and CRC incidence and mortality among initially healthy individuals. In exploratory analyses, GlycA was associated with more advanced stage tumors. Tumor stage is considered the strongest prognostic factor in CRC. Higher GlycA levels were also associated with increased risk of proximal tumors, which are more difficult to detect and prevent by routine colonoscopy.[25]

The model of glycosylation-dependent promotion of tumor progression has developed in conjunction with clinicopathological studies.[17] Increased expression of some glycosyl moieties promotes invasion and metastasis, leading to shorter patient survival rates, whereas expression of other glycosyl epitopes suppresses tumor progression, resulting in higher survival rates.[26] Inflammation and immune function are united in the pathogenesis of cancers[27, 28] and underlie the mechanistic importance of protein post-translational glycosylation in the pathogenesis of CRC.[29]

Glycosylated acute phase proteins undergo dynamic changes in concentration in response to systemic tissue injury and may be exploited as tumor markers.[30] Acute phase proteins are relevant to many key biological processes including cell adhesion, molecular trafficking and clearance, signal transduction, modulation of the innate immune system and inflammation.[31–33] Prior research has shown that GlycA is associated with cardiometabolic diseases[34, 35] and autoimmune diseases.[36] The commonality of these conditions is that they are driven by inflammation.

From the perspective of CRC, prior work has demonstrated that acute phase proteins may provide additional information when combined with CEA. Ward et al. reported that rises in GlycA acute phase proteins, α1-antitrypsin, α1-acid glycoprotein, and haptoglobin, of postoperative CRC patients were associated with metastases or recurrent cancer;[37] in healthy individuals, α1-antitrypsin, haptoglobin, and α1-acid glycoprotein profiles were stable over time.[37] Furthermore, a model that included preoperative α1-antitrypsin, and α1-acid glycoprotein blood levels considerably improved the predictive value of the model attained from using CEA levels alone.[37] Additionally, serial measurements of several acute phase proteins strengthened the observed association between CEA and prognosis for monitoring postoperative CRC patients.[37] Our study is in agreement with a prior study of colorectal cancer at the time of surgery that observed no significant correlation between CRP, α1-antitrypsin, CEA and the stage of the disease, but significant correlations were observed between the α1-acid glycoprotein (CA 19–9) and stage of the disease.[38]

Similar relationships with CRC were noted in a prior study examining human plasma N-glycans measured with a different technique (high-performance liquid chromatography).[17] These observations along with the chemical characteristics of GlycA suggest that an important component of the risk predicted by GlycA is related to systemic inflammation that is not completely measured by the other inflammatory biomarkers (hsCRP, fibrinogen, sICAM-1). As GlycA measures the N-acetyl glycan moieties on circulating blood glycoproteins commonly found on acute phase proteins, it may be identifying another aspect of risk related to inflammation. The correlation between GlycA and CRC risk factors such as smoking, BMI, physical activity, and red meat intake, highlight a role for modifiable lifestyle risk factors in the expression of protein glycans that produce the GlycA signal. In the WHS study population, no effect modification was observed for aspirin, NSAIDs, or vitamin E to suggest a possible intervention pathway for these agents on GlycA. Alternatively, GlycA may be reflecting alterations in the glycosylation pathway involved in the pathogenesis of CRC.

Strengths of our study include the long prospective follow-up (median 19 years in WHS and 11 years in MESA) of participants (27,495 in WHS and another 6,784 in MESA), well-characterized CRC pathology with standardized ascertainment of incident CRC and CRC mortality in WHS, detailed information about CRC risk factors, extensive biomarker phenotyping, and the use of independent derivation and validation cohorts. Limitations to our study interpretations exist. First, only baseline blood samples were available. Yet, a previous study with repeated measures of GlycA showed that GlycA may be elevated for over a decade.[24] Second, the observational nature of this study precludes our ability to identify mechanisms for the observed association of GlycA with increased risk of incident CRC and CRC mortality. Third, we did not have CEA measurements but a previous study of acute phase proteins and incident CRC did not show significant correlations between serum CEA, α1-antitrypsin, and CRP levels with the stage of disease.[38] Fourth, GlycA is related to a number of inflammatory conditions,[35, 36] but the GlycA association with CRC was stronger than CRP or other inflammatory biomarkers in our study. Metabolic syndrome[35] and other inflammatory conditions[39] associated with elevated GlycA have been linked to CRC.[40] Finally, the lack of information on the frequency of colonoscopies may contribute to lead time bias. Furthermore, MESA did not exclude participants with pre-existing cancer or history of treatment for cancer.

## Conclusions

In conclusion, we have identified a novel association between elevated baseline levels of GlycA, an NMR-measured biomarker of circulating N-linked glycoprotein acetyls on several acute phase proteins, and incident CRC and mortality. GlycA may be either a complementary biomarker of systemic inflammation or may represent risk related to alternate disease pathways. Future studies should evaluate the role of GlycA in conjunction with standard colon cancer screening tools. Additional studies are needed to explore the range of potential for GlycA in the prevention and prognostication of CRC.

## Supporting Information

S1 File Supplemental methods. Discovery Study Population, Laboratory Measurements, Ascertainment of CRC Cases and Death, Replication Cohort, Models for Statistical Analyses, Figures, and Tables.
**Fig A in S1 File.** Schematic example of tri-antennary N-acetyl linked glycan chain, with N-acetylglucosamine (GlcNAc) contributing to the GlycA signal (red box).
**Fig B in S1 File.** Cohort Diagrams for Women’s Health Study and Multi-ethnic Study of Atherosclerosis
**Table A in S1 File.** Spearman correlation coefficients (r) between GlycA and acute phase reactants in WHS and MESA
**Table B in S1 File.** WHS colorectal cancer incidence and mortality by quartiles of baseline GlycA, hsCRP, sICAM-1, and fibrinogen
**Table C in S1 File.** Association of GlycA with incident colorectal cancer and colorectal cancer death after additionally adjusting for inflammatory biomarkers
**Table D in S1 File.** Baseline clinical and biochemical variables by GlycA tertile in MESA
**Table E in S1 File.** MESA colorectal cancer incidence and mortality by tertiles of GlycA.
(DOCX)
