# Supplementary material for: Association of N-Linked Glycoprotein Acetyls and Colorectal Cancer Incidence and Mortality
Source: PLoS One. 2016 Nov 30;11(11):e0165615. doi: 10.1371/journal.pone.0165615 (PMC5130185; doi:10.1371/journal.pone.0165615)
Supplement: S1 File — Discovery Study Population, Laboratory Measurements, Ascertainment of CRC Cases and Death, Replication Cohort, Models for Statistical Analyses, Figures, and Tables. Fig A in S1 File. Schematic example of tri-antennary N-acetyl linked glycan chain, with N-acetylglucosamine (GlcNAc) contributing to the GlycA signal (red box). Fig B in S1 File. Cohort Diagrams for Women’s Health Study and Multi-ethnic Study of Atherosclerosis Table A in S1 File. Spearman correlation coefficients (r) between GlycA and acute phase reactants in WHS and MESA Table B in S1 File. WHS colorectal cancer incidence and mortality by quartiles of baseline GlycA, hsCRP, sICAM-1, and fibrinogen Table C in S1 File. Association of GlycA with incident colorectal cancer and colorectal cancer death after additionally adjusting for inflammatory biomarkers Table D in S1 File. Baseline clinical and biochemical variables by GlycA tertile in MESA Table E in S1 File. MESA colorectal cancer incidence and mortality by tertiles of GlycA. (DOCX) [file pone.0165615.s001.docx]

**SMETHODS**

**Discovery Study Population:** Of those alive at the end of trial in 2004, 33,682 (85%) consented to continue in the post-trial follow-up of participants. Prior to randomization, blood was requested (but not required) from participants. Women who did and did not donate blood were similar on a wide range of variables related to cancer.[[1](#_ENREF_1)] Anthropometric, lifestyle, and dietary data were derived from the baseline questionnaire. The validity and reproducibility of the semi-quantitative food-frequency questionnaire (FFQ) and other self-reported variables have been described previously.[[2-4](#_ENREF_2)] Written informed consent was obtained from each participant.

**Laboratory Measurements:** The plasma 400 Hz ^1^H NMR GlycA signal at 2.00 ppm was quantified using deconvolution software to measure a subset of mobile N-acetylglucosamine (GlcNAc) residues on the bi-, tri-, and tetra-antennary glycan branches of circulating glycoproteins (see eFigure 1 in Supplement). Comparison of GlycA to the resonance spectra of targeted proteins identified the predominant constituents of GlycA as α1-acid glycoprotein, haptoglobin, α1-antitrypsin, α1-antichymotrypsin, and transferrin.[[5](#_ENREF_5),[6](#_ENREF_6)] Notably, other acute phase proteins (including fibrinogen, α2-macroglobulin, sICAM-1, and CRP), do not contribute to the GlycA signal.[[6](#_ENREF_6)] GlycA was measured on blood samples obtained at baseline for WHS and MESA that had been collected in EDTA tubes and stored in vapor‐phase liquid nitrogen (−170°C for WHS and −70°C for MESA). LipoScience (now LabCorp; Raleigh, NC) performed the GlycA measurements as an adjunct to the LipoProfile-3 assay. Reported GlycA intra and inter-assay coefficients of variation are 1.9 and 2.6% µmol/L.[[6](#_ENREF_6)]

In WHS, hsCRP was measured by an immunoturbidimetric assay with reagents and calibrators from Denka Seiken (Tokyo, Japan).[[7](#_ENREF_7)] sICAM-1 was measured using an enzyme-linked immunosorbent assay (R&D Systems, Minneapolis, MN), and fibrinogen was measured using an immunoturbidimetric assay (Kamiya Biomedical, Seattle, WA).[[8](#_ENREF_8)]

**Ascertainment of CRC Cases and Death:** In WHS (median follow-up 19 years), CRC cases were ascertained via annual follow-up questionnaires, letters, and telephone calls. Following written informed consent, medical records were obtained and reviewed by a blinded Endpoints Committee that adjudicated all reported endpoints including CRC based on predefined criteria.[[1](#_ENREF_1)] Deaths of participants were identified by reports from family members, postal authorities, and a search of the National Death Index (NDI). Medical records were reviewed to confirm CRC cases. Then the events were coded with ICD codes. Morbidity and mortality follow-up were 97.2 and 99.4% complete, respectively.[[9](#_ENREF_9)]

**Replication Cohort:** MESA CRC cases (median follow-up 11 years) were identified from hospital records by ICD-9 and ICD-10 codes along with NDI for CRC deaths. The following ICD codes were used to identify these participants:  ICD-9:  153.* (Malignant neoplasm of colon), 154.* (Malignant neoplasm of rectum rectosigmoid junction and anus), ICD-10: C18.* (Malignant neoplasm of colon), C19.* (Malignant neoplasm of rectosigmoid junction), C20.* (Malignant neoplasm of rectum), C21.* (Malignant neoplasm of anus and anal canal). Cancer outcome information was obtained from the records collected during investigation of MESA endpoints of interest (myocardial infarction, angina, congestive heart failure, peripheral vascular disease, stroke, transient ischemic attack, revascularization, and deaths due to cardiovascular disease[CVD]). Only CVD events undergo rigorous physician review for endpoint adjudication.  Non-CVD events (such as CRC) do not undergo this physician review and are defined expressly based on the ICD-9/10 codes obtained from discharge summaries (all hospitalizations).[[10](#_ENREF_10)]

**Models for Statistical Analyses**

In WHS, model 1 adjusted for trial treatment assignments and age. Model 2 additionally included race, family history of CRC, alcohol, exercise, smoking, menopausal status, postmenopausal hormone use (never, past, current); healthy eating index (Alternative Healthy Eating Index [AHEI], which is based on foods and nutrients predictive of chronic disease risk),[[11](#_ENREF_11)] multivitamin use, intake of red meat (servings/day), total vegetable and fruits (servings/day), supplemental and dietary calcium, fiber (grams/day), total calories (kcal/day); history of colonic polyps, body mass index (BMI), and hemoglobin A1C. The third model included model 2 variables plus one of the markers of systemic inflammation (natural log transformed [ln]hsCRP, fibrinogen, or sICAM-1). Given the smaller number of events in the replication cohort (MESA), risk of CRC incidence and mortality were modeled with two models: model 1 adjusted for age, sex, and race/ethnicity; model 2 included model 1 variables and adjusted for BMI, exercise (total intentional exercise MET-hrs/wk), smoking, alcohol (drinks/week), and family history of cancer.

References

1. Zhang SM, Buring JE, Lee IM, Cook NR, Ridker PM (2005) C-reactive protein levels are not associated with increased risk for colorectal cancer in women. Ann Intern Med 142: 425-432.

2. Willett WC, Reynolds RD, Cottrell-Hoehner S, Sampson L, Browne ML (1987) Validation of a semi-quantitative food frequency questionnaire: comparison with a 1-year diet record. J Am Diet Assoc 87: 43-47.

3. Willett WC, Sampson L, Stampfer MJ, Rosner B, Bain C, et al. (1985) Reproducibility and validity of a semiquantitative food frequency questionnaire. Am J Epidemiol 122: 51-65.

4. Salvini S, Hunter DJ, Sampson L, Stampfer MJ, Colditz GA, et al. (1989) Food-based validation of a dietary questionnaire: the effects of week-to-week variation in food consumption. Int J Epidemiol 18: 858-867.

5. Bell JD, Brown JC, Nicholson JK, Sadler PJ (1987) Assignment of resonances for 'acute-phase' glycoproteins in high resolution proton NMR spectra of human blood plasma. FEBS Lett 215: 311-315.

6. Otvos JD, Shalaurova I, Wolak-Dinsmore J, Connelly MA, Mackey RH, et al. (2015) GlycA: A Composite Nuclear Magnetic Resonance Biomarker of Systemic Inflammation. Clin Chem 61: 714-723.

7. Ridker PM, Rifai N, Rose L, Buring JE, Cook NR (2002) Comparison of C-reactive protein and low-density lipoprotein cholesterol levels in the prediction of first cardiovascular events. N Engl J Med 347: 1557-1565.

8. Mora S, Lee IM, Buring JE, Ridker PM (2006) Association of physical activity and body mass index with novel and traditional cardiovascular biomarkers in women. JAMA 295: 1412-1419.

9. Cook NR, Lee IM, Zhang SM, Moorthy MV, Buring JE (2013) Alternate-day, low-dose aspirin and cancer risk: long-term observational follow-up of a randomized trial. Ann Intern Med 159: 77-85.

10. Carter CE, Gansevoort RT, Scheven L, Heerspink HJ, Shlipak MG, et al. (2012) Influence of urine creatinine on the relationship between the albumin-to-creatinine ratio and cardiovascular events. Clin J Am Soc Nephrol 7: 595-603.

11. Chiuve SE, Fung TT, Rimm EB, Hu FB, McCullough ML, et al. (2012) Alternative dietary indices both strongly predict risk of chronic disease. J Nutr 142: 1009-1018.

**Table A. Spearman correlation coefficients (r) between GlycA and acute phase reactants in WHS and MESA***

|  | **GlycA, µmol/L** | | | |
| --- | --- | --- | --- | --- |
|  | **WHS** | | **MESA** | |
|  | r | N | r | N |
| Fibrinogen | 0.46 | 27,308 | 0.47 | 6,753 |
| hsCRP | 0.61 | 27,458 | 0.55 | 6,750 |
| ICAM-1 | 0.30 | 27,297 | 0.20 | 2,617 |

**Abbreviations:** hsCRP = high-sensitivity C-reactive protein, ICAM-1=soluble intracellular adhesion molecule 1, WHS = Women’s Health Study, MESA=Multi-Ethnic Study of Atherosclerosis, N = number of individuals, and r = correlation.

*All correlations p<0.0001.

**Table B. Baseline clinical and biochemical variables by GlycA tertile in MESA^a^**

| **Tertiles of GlycA, umol/L** | | | |
| --- | --- | --- | --- |
|  | <351  (n=2265) | 351-404  (n=2285) | >405  (n=)2234 |
| **Incident colorectal cancer cases** | 18 | 19 | 33 |
| **GlycA, mean (SD), µmol/L** | 317.3 (25.4) | 377.2 (15.1) | 451.5 (42.1) |
| **Age, years, mean (SD)^b^** | 61.7 (10.4) | 62.6 (10.2) | 62.2 (10.1) |
| **Women, %** | 39.5 | 52.3 | 66.8 |
| **Race/ethnicity, (%)** |  |  |  |
| **Caucasian** | 37.3 | 40.2 | 38.1 |
| **Black** | 25.7 | 26.3 | 31.0 |
| **Chinese** | 18.1 | 11.7 | 5.7 |
| **Hispanic** | 18.9 | 21.8 | 25.3 |
| **Body mass index (kg/m^2^)** | 26.7 (4.7) | 28.2 (5.2) | 30.1 (6.0) |
| **Physical activity (MET-hr/wk)** | 29.7 (39.9) | 25.5 (41.4) | 22.5 (35.1) |
| **hsCRP, mg/L** | 1.6 (2.0) | 3.1 (3.9) | 6.8 (8.4) |
| **Smoking status, (%)** |  |  |  |
| **Current** | 9.4 | 12.1 | 17.6 |
| **Past** | 38.7 | 36.7 | 34.7 |
| **Never** | 52.0 | 51.2 | 47.8 |
| **Alcohol (no. drinks/week)^c^** | 4.3 (8.8) | 3.8 (7.7) | 4.0 (9.1) |
| **Family history of cancer,%^c^** | 54.4 | 56.7 | 57.8 |

**Abbreviations**: BMI=body mass index, hsCRP=high sensitivity C-reactive protein, MET-hrs/wk=metabolic equivalent hours per week. Data presented as mean (standard deviation) otherwise as percent where indicated.

^a^ P value <0.001 unless otherwise indicated.

^b^ P value=0.009

^c^ P value >0.05

|  | **Tertiles of GlycA, umol/L** | | | |  |  |
| --- | --- | --- | --- | --- | --- | --- |
|  | **Q1** | **Q2** | **Q3** | **P Linear trend** | **Per 1 SD^*^** | **P Value** |
|  | <351 | 351-404 | >404 |  |  |  |
| **Incident CRC, N cases/total** | 18/2265 | 19/2285 | 33/2234 |  |  |  |
| **Incident Rate per 1,000 person-years** | 0.76 | 0.80 | 1.48 |  |  |  |
| **CRC deaths, N cases/total** | 4/2265 | 6/2285 | 13/2234 |  |  |  |
| **Death Rate per 1,000 person-years** | 0.17 | 0.25 | 0.58 |  |  |  |
| **Incident CRC, Model 1** | 1.0 (ref) | 0.99 (0.52-1.91) | 1.87 (1.03-3.40) | 0.02 | 1.32 (1.06-1.65) | 0.01 |
| **Incident CRC, Model 2** | 1.0 (ref) | 0.98 (0.50-1.91) | 1.57 (0.83-2.97) | 0.12 | 1.21 (0.95-1.55) | 0.12 |
| **CRC death,**  **Model 1** | 1.0 (ref) | 1.18 (0.33-4.25) | 2.63 (0.83-8.39) | 0.06 | 1.54 (1.06-2.23) | 0.02 |
| **CRC death,**  **Model 2** | 1.0 (ref) | 1.39 (0.44-5.66) | 2.41 (0.64-9.14) | 0.14 | 1.34 (0.88-2.03) | 0.17 |

**Table C. MESA colorectal cancer incidence and mortality by tertiles of GlycA^*^**

**Abbreviations**: BMI=body mass index, MET-hrs/wk=metabolic equivalent hours per week.

^*^ Model 1: adjusted for age, race, gender. Model 2: Model 1+BMI, exercise (total intentional exercise MET-hr/wk), smoking, alcohol ( number drinks per week), family history of cancer.

SD represents standard deviation (62 µmol/L for GlycA)

| **GlycA** |  |  |  |  |  |  |  |
| --- | --- | --- | --- | --- | --- | --- | --- |
| **Range,**  **µmol/L** | <326 | 327-369 | 370-416 | >416 |  |  |  |
| **Incident CRC, model 1** | 1.0 (ref) | 1.08 (0.77-1.53) | 1.29 (0.93-1.79) | 1.83 (1.35-2.50) | <0.0001 | 1.27 (1.14-1.41) | <0.0001 |
| **Incident CRC, model 2** | 1.0 (ref) | 1.05 (0.74-1.51) | 1.24 (0.89-1.77) | 1.55 (1.09-2.20) | 0.006 | 1.19 (1.06-1.35) | 0.004 |
| **CRC death, model 1** | 1.0 (ref) | 1.01 (0.54-1.88) | 1.21 (0.67-2.19) | 1.74 (1.00-3.03) | 0.02 | 1.33 (1.10-1.61) | 0.003 |
| **CRC death, model 2** | 1.0 (ref) | 0.92 (0.47-2.80) | 1.26 (0.67-2.37) | 1.46 (0.77-2.77) | 0.14 | 1.24 (1.00-1.54) | 0.05 |
| **Fibrinogen** |  |  |  |  |  |  |  |
| **Range, mg/dL** | <308 | >308-351 | >351-403 | >403 |  |  |  |
| **Incident CRC, Model 1** | 1.0 (ref) | 1.05 (0.74-1.49) | 1.17 (0.83-1.64) | 1.59 (1.16-2.20) | 0.001 | 1.11 (0.99-1.24) | 0.08 |
| **Incident CRC, Model 2** | 1.0 (ref) | 1.01 (0.71-1.44) | 1.04 (0.73-1.48) | 1.31 (0.92-1.85) | 0.09 | 1.01 (0.90-1.14) | 0.89 |
| **CRC death, Model 1** | 1.0 (ref) | 1.12 (0.52-2.39) | 2.12 (1.09-4.13) | 2.39 (1.24-4.61) | 0.002 | 1.26 (1.03-1.55) | 0.02 |
| **CRC death, Model 2** | 1.0 (ref) | 0.97 (0.45-2.11) | 1.79 (0.90-3.57) | 1.81 (0.90-3.67) | 0.04 | 1.12 (0.89-1.41) | 0.33 |
| **HsCRP** |  |  |  |  |  |  |  |
| **Range, mg/L** | <0.8 | >0.8-2 | >2-4.4 | >4.4 |  |  |  |
| **Incident CRC, Model 1** | 1.0 (ref) | 0.85 (0.61-1.18) | 0.99 (0.72-1.36) | 1.16 (0.85-1.57) | 0.11 | 1.09 (0.98-1.23) | 0.13 |
| **Incident CRC, Model 2** | 1.0 (ref) | 0.79 (0.56-1.11) | 0.84 (0.60-1.18) | 0.88 (0.61-1.26) | 0.93 | 0.99 (0.86-1.14) | 0.88 |
| **CRC death, Model 1** | 1.0 (ref) | 1.02 (0.55-1.91) | 1.30 (0.72-2.35) | 1.30 (0.72-2.35) | 0.33 | 1.11 (0.90-1.38) | 0.32 |
| **CRC death, Model 2** | 1.0 (ref) | 1.00 (0.53-1.89) | 1.13 (0.60-2.15) | 1.08 (0.54-2.16) | 0.82 | 1.02 (0.79-1.31) | 0.90 |
| **sICAM-1** |  |  |  |  |  |  |  |
| **Range, ng/mL** | 74-301 | >301-343 | >343-395 | >395 |  |  |  |
| **Incident CRC, Model 1** | 1.0 (ref) | 1.07 (0.76-1.51) | 1.24 90.89-1.72) | 1.40 (1.02-1.94) | 0.02 | 1.10 (0.98-1.23) | 0.12 |
| **Incident CRC, Model 2** | 1.0 (ref) | 1.04 (0.73-1.47) | 1.13 (0.80-1.58) | 1.16 (0.82-1.65) | 0.35 | 1.01 (0.89-1.15) | 0.84 |
| **CRC death, Model 1** | 1.0 (ref) | 0.99 (0.53-1.83) | 0.88 (0.47-1.65) | 1.48 (0.84-2.60) | 0.11 | 1.20 (0.98-1.47) | 0.08 |
| **CRC death, Model 2** | 1.0 (ref) | 0.93 (0.50-1.73) | 0.79 (0.42-1.49) | 0.99 (0.53-1.84) | 0.99 | 1.02 (0.81-1.28) | 0.89 |

**Abbreviations:** hsCRP=high sensitivity C-reactive protein, sICAM-1 = soluble intracellular adhesion molecule 1

* Model 1: Hazard ratio from Cox regression models adjusted for age and trial treatment assignment

Model 2: Hazard ratio from Cox regression models adjusted for age, trial treatment assignment, race, family history of colorectal cancer, alcohol, exercise, smoking, menopausal status, postmenopausal hormone use; alternative healthy eating index, multivitamin use; intake of red meat, vegetables and fruits, supplemental and dietary calcium, fiber, total calories, history of polyps, body mass index, and hemoglobin A1c.

SD represents standard deviation for baseline inflammatory biomarkers in WHS (1.20 mg/L for hsCRP, 0.22 ng/mL for sICAM-1, 0.22 mg/dL for fibrinogen);hsCRP, fibrinogen, and sICAM-1 were log transformed.

|  |  | **Quartiles of Baseline GlycA** | |  |  |  |  |
| --- | --- | --- | --- | --- | --- | --- | --- |
|  | Q1 | Q2 | Q3 | Q4 | P linear trend | 1-SD | P value |
|  |  |  |  |  |  |  |  |
| Range, umol/L | <326 | 327-369 | 370-416 | >416 |  |  |  |
| CRC Incidence |  |  |  |  |  |  |  |
| Model 2 | 1.0 (ref) | 1.05 (0.73-1.50) | 1.23 (0.87-1.75) | 1.55 (1.09-2.20) | 0.006 | 1.19 (1.06-1.35) | 0.004 |
| Model 2 + fibrinogen | 1.0 (ref) | 1.07 (0.74-1.53) | 1.26 (0.88-1.79) | 1.62 (1.12-2.35) | 0.005 | 1.22 (1.07-1.39) | 0.003 |
| Model 2 +hsCRP | 1.0 (ref) | 1.09 (0.76-1.57) | 1.33 (0.93-1.92) | 1.76 (1.20-2.60) | 0.002 | 1.27 (1.10-1.45) | 0.0009 |
| Model 2 + sICAM-1 | 1.0 (ref) | 1.05 (0.73-1.51) | 1.24 (0.87-1.75) | 1.56 (1.09-2.23) | 0.006 | 1.20 (1.06-1.35) | 0.004 |
| CRC Death |  |  |  |  |  |  |  |
| Model 2 | 1.0 (ref) | 0.92 (0.47-1.80) | 1.26 (0.67-2.36) | 1.46 (0.77-2.76) | 0.15 | 1.24 (1.00-1.54) | 0.05 |
| Model 2 + fibrinogen | 1.0 (ref) | 0.90 (0.46-1.77) | 1.21 (0.64-2.30) | 1.38 (0.70-2.71) | 0.24 | 1.23 (0.97-1.56) | 0.09 |
| Model 2+hsCRP | 1.0 (ref) | 0.95 (0.48-1.86) | 1.33 (0.69-2.56) | 1.54 (0.76-3.14) | 0.15 | 1.31 (1.01-1.68) | 0.04 |
| Model 2+ sICAM-1 | 1.0 (ref) | 0.92 (0.47-1.80) | 1.26 (0.67-2.36) | 1.47 (0.77-2.79) | 0.15 | 1.25 (1.00-1.56) | 0.049 |

**Table E. Association of GlycA with incident colorectal cancer and colorectal cancer death after additionally adjusting for inflammatory biomarkers^*^**

**Abbreviations:** hsCRP=high sensitivity C-reactive protein, sICAM-1 = soluble intracellular adhesion molecule 1

^*^ Model 2: Hazard ratio from Cox regression models adjusted for age, trial treatment assignment, race, family history of colorectal cancer, alcohol, exercise, smoking, menopausal status, postmenopausal hormone use; alternative healthy eating index, multivitamin use; intake of red meat, vegetables and fruits, supplemental and dietary calcium, fiber, total calories, history of polyps, body mass index, and hemoglobin A1c.

SD represents standard deviation for baselineGlycA in WHS (68 µmol/L for GlycA); hsCRP, fibrinogen, and sICAM-1 were log transformed.

**Fig A : Schematic example of tri-antennary N-acetyl linked glycan chain, with N-acetylglucosamine (GlcNAc) contributing to the GlycA signal (red box).** GlycA identifies bi-, tri-, and tetra-antennary N-linked glycan chains with β1→2 and β1→6 mannose-GlcNAc linkage. Asparagine represents the universal amino acid site for N-glycosylation. (Modified from Otvos *et al*. *Clinical Chemistry* 2015.[^14^](#_ENREF_14))

Cohort Diagrams for Women’s Health Study and Multi-ethnic Study of Atherosclerosis

**
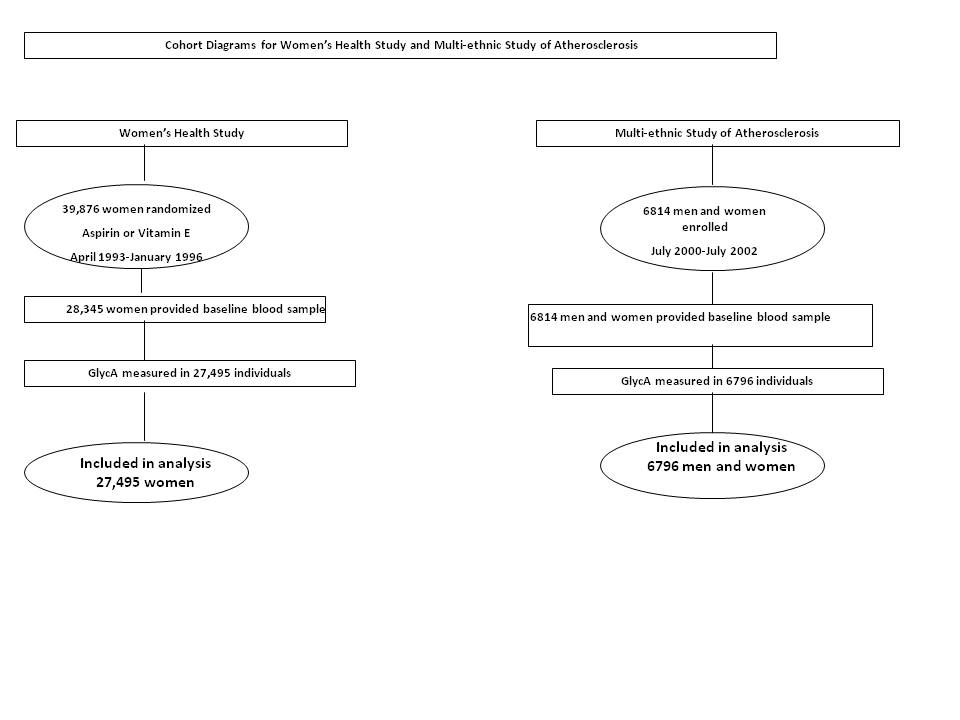
Fig B.** Cohort Diagrams for Women’s Health Study and Multi-ethnic Study of Atherosclerosis
